# Progesterone supplementation for HIV-positive pregnant women on protease inhibitor-based antiretroviral regimens (the ProSPAR study): a study protocol for a pilot randomized controlled trial

**DOI:** 10.1186/s40814-016-0087-6

**Published:** 2016-08-12

**Authors:** Kaitlin Siou, Sharon L. Walmsley, Kellie E. Murphy, Janet Raboud, Mona Loutfy, Mark H. Yudin, Michael Silverman, Noor N. Ladhani, Lena Serghides

**Affiliations:** 1 Toronto General Research Institute, Toronto, Canada; 2 Toronto General Hospital, Toronto, Canada; 3 University of Toronto, Toronto, Canada; 4 Mount Sinai Hospital, Toronto, Canada; 5 Maple Leaf Medical Clinic, Toronto, Canada; 6 Women’s College Research Institute, Toronto, Canada; 7 St. Michael’s Hospital, Toronto, Canada; 8 St. Joseph’s Health Care London, London, Canada; 9 University of Western Ontario, London, Canada; 10 Sunnybrook Health Sciences Centre, Toronto, Canada

**Keywords:** HIV, Pregnancy, Protease inhibitors, Progesterone supplementation, Low birth weight, Preterm birth, RCT, Feasibility, Pilot study

## Abstract

**Background:**

In Canada, the majority of HIV-positive pregnant women receive combination antiretroviral therapy that includes a ritonavir-boosted protease inhibitor to prevent mother-to-child HIV transmission. However, protease inhibitor-based combination antiretroviral therapy has been associated with increased rates of preterm, low birth weight, and small for gestational age births. Our previous experimental findings demonstrate that protease inhibitor use during pregnancy is associated with decreased progesterone levels that correlate with fetal growth, and that progesterone supplementation can improve protease inhibitor-induced fetal growth restriction. We hypothesize that HIV-positive pregnant women who receive protease inhibitor-based combination therapy may also benefit from progesterone supplementation during pregnancy.

**Methods/design:**

In order to test this hypothesis, we have designed an open-label, multi-centre, randomized controlled (parallel group) pilot trial. The initial goal of this trial is to test feasibility and acceptability of our intervention. Forty HIV-positive pregnant women who are either on, or intending to start or switch to a boosted protease inhibitor-based combination antiretroviral regimen will be enrolled from six sites across Ontario, Canada. Twenty-five women will be randomized to self-administer natural progesterone (Prometrium, 200 mg) vaginally every night starting between gestational week 16 and 24 until week 36, and 15 women will be randomized to no intervention. While the participants and treating physicians will not be blinded, the laboratory personnel performing the biochemical and morphological evaluations will be blinded to ensure unbiased evaluation. The primary outcome of the pilot study is the feasibility of enrolment as measured by the recruitment rate and patient-reported reasons to decline participation. Secondary outcomes in participants include safety, acceptability, and adherence to progesterone supplementation.

**Discussion:**

Given the safety of intravaginal progesterone and its current use in the general obstetrical population to prevent recurrent preterm delivery, this pilot study will provide data to determine the feasibility of a larger randomized controlled trial to assess the impact of this intervention on improving neonatal health in the context of HIV-positive pregnancies.

**Trial registration:**

ClinicalTrials.gov, NCT02400021

## Background

### Preventing perinatal HIV transmission

Combination antiretroviral therapy (cART) is recommended for all HIV-positive pregnant women for the prevention of mother-to-child HIV transmission and for optimal maternal health. Given the results of recent studies on the health benefits of early cART initiation [1], HIV-positive women are increasingly initiating cART before conception or early in pregnancy. Furthermore, data suggest that earlier initiation of therapy in pregnancy is associated with lower rates of perinatal transmission consequent to prolonged suppression of viral load during pregnancy [2–4]. Since the safety of some newer agents in pregnancy is unknown, it is typical practice to switch cART to one of the guideline-recommended combinations prior to a planned pregnancy. The majority of HIV-positive pregnant women in Canada receive a cART regimen that includes a boosted protease inhibitor (PI) [5].

### The potential risk of protease inhibitor-based combination antiretroviral therapy in pregnancy

Although controversial and often complicated by multiple confounders, data from numerous studies suggest an association between PI-based cART and preterm, low birth weight, and small for gestational age births [6–29]. Preterm birth and low birth weight are significant factors contributing to infant morbidity and mortality, and have been associated with severe short-term and long-term adverse health and social outcomes including increased risk of developmental delay in children [30–35], and higher risk for developing chronic ailments such as diabetes, heart disease, and asthma later in life [36–39].

### Declining progesterone levels as a potential mechanism for the impact of protease inhibitors on birth outcomes

We have previously reported experimental findings demonstrating that PI use during pregnancy is associated with decreased progesterone levels that correlate with fetal growth, and that progesterone supplementation can improve PI-induced fetal growth restriction [40]. Exposure of a human placental cell line to PIs (atazanavir, lopinavir, ritonavir), but not nucleoside reverse transcriptase inhibitors (NRTIs; zidovudine, lamivudine) or a non-NRTI (NNRTI; nevirapine), resulted in decreased progesterone production. In an animal model of pregnancy, lower progesterone levels were detected in mice exposed to PI-based cART (ritonavir-boosted lopinavir) but not in those exposed only to the NRTI backbone. Moreover, the change in progesterone levels was associated with smaller fetuses and other adverse birth outcomes. To establish a direct relationship between progesterone levels and birth outcomes, PI-based cART exposed mice were supplemented with natural progesterone throughout their pregnancy. Progesterone supplementation significantly increased fetal weight in these mice.

Consistent with these data, we have also observed lower progesterone levels at gestational weeks 25–28 in HIV-positive pregnant women exposed to PI-based cART compared to HIV-negative pregnant women. Progesterone levels in the HIV-positive women were significantly correlated with birth weight percentile [40].

### Study rationale and objectives

In the general population, progesterone supplementation is widely used in pregnancy for the prevention of recurrent preterm birth [41, 42]. Progesterone supplementation in pregnancy is well tolerated and considered safe [41, 43–47]. HIV-positive women have higher rates of preterm birth and low birth weight that may be magnified by the use of PIs. Based on our pre-clinical and clinical findings, we hypothesized that HIV-positive pregnant women who receive PI-based cART might also benefit from progesterone supplementation during their pregnancy.

The primary objective of this pilot study is to determine the feasibility of enrolling HIV-positive women on PI-based cART during pregnancy into a randomized clinical trial of progesterone supplementation. Secondary objectives include determining preliminary information on the safety, acceptability, compliance, and barriers to adherence to progesterone supplementation during pregnancy for HIV-positive women. The data will also be used to establish estimates of the standard deviation and the intra-patient correlation coefficient of progesterone levels during pregnancy in order to inform the design of a larger clinical endpoint study.

## Methods/design

### Study design and intervention

**Fig. 1:**
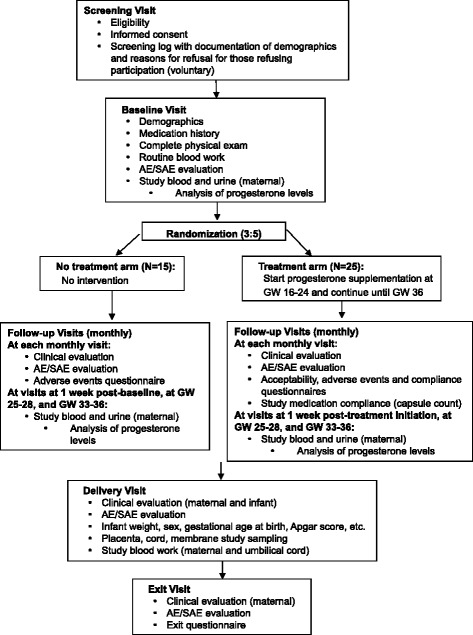
Schematic of study design

This is an open-label, multi-centre, controlled pilot trial in which 40 HIV-positive pregnant women on PI-based cART will be randomized into two groups with an allocation ratio of 5:3. Group A will self-administer intravaginal progesterone (Prometrium, 2 × 100 mg capsules) once every night before bed starting between gestational week (GW) 16–24 until GW36, while group B will receive no intervention (see Fig. 1 for a schematic of the study design).

### Study setting

Recruitment will take place at six study sites in Ontario, Canada: the Toronto General Hospital Immunodeficiency clinic, the obstetrical clinic at Mount Sinai Hospital, the obstetrical clinic at St. Michael’s Hospital, the Maple Leaf Medical clinic, the obstetrical clinic at Sunnybrook Health Sciences Centre, and the Infectious Diseases Care Program at St. Joseph’s Health Care London. All HIV-positive, pregnant women attending or referred to one of the study centers during the trial will be assessed for eligibility and participation, and those who consent to the study will be followed at the site at which recruitment occurs.

### Participant eligibility criteria

To be considered for enrollment in this pilot study, the following inclusion criteria must be fulfilled:

1. Female; 16 years or older
2. Documented HIV-1 infection
3. On stable, or initiating, cART containing either ritonavir-boosted lopinavir (LPV/r), ritonavir-boosted atazanavir (ATZ/r), or ritonavir-boosted darunavir (DRV/r)
4. Pregnant up to 24 weeks gestational age
5. Singleton pregnancy
6. Ability to give informed consent

Participants are excluded based on the following criteria:

1. Hypersensitivity or allergy to soya or peanut (non-active ingredient supplement in Prometrium)
2. Contraindications to intravaginal progesterone use including:
  - Documented hypersensitivity to Prometrium
  - Active or history of breast cancer
  - Active or history of arterial thromboembolic disease (e.g., stroke, myocardial infarction, coronary heart disease)
  - Active or history of venous thromboembolism (e.g., deep venous thrombosis or pulmonary embolism) or active thrombophlebitis
  - Any prior neoplasia, except for skin
  - Abnormal vaginal bleeding
3. Known lethal fetal anomaly
4. Any contraindication to continuation of pregnancy
5. Inability to communicate in English
6. Prior participation in this trial
7. Prior experience with intravaginal progesterone administration

### Outcomes

The primary outcome is feasibility. The proportion of HIV-positive, pregnant women who are approached and screened but do not meet eligibility criteria will be recorded, as well as the proportion of women who meet eligibility criteria but decline to participate. Data will be collected on the gestational age at screening and enrollment. Data will also be collected on the reasons for non-participation. This information will be used to determine the number of centers that will be required for timely recruitment of a larger randomized control trial (RCT). A consent rate of less than 60 % among women that meet eligibility criteria would be grounds for not moving forward with a larger RCT in this setting.

Secondary outcomes include safety, acceptability, compliance, and barriers to adherence. Safety and tolerability will be assessed through questionnaires administered to participants during the study, and by recording adverse events (AE) using the Division of AIDS (DAIDS) Table for Grading the Severity of Adult and Pediatric AEs and Addendum 1 Female Genital Grading Table for Use in Microbicide Studies [48]. AEs of all grades will be recorded, and the proportion of participants who report AEs will be compared between study arms. Any grade 4 AE or severe adverse event (SAE) attributed to progesterone supplementation would be grounds for not moving forward with a larger RCT. Standardized questions will capture group A’s experience with progesterone self-administration and information on local irritation or discomfort. Study acceptability will be measured by capturing whether participants are willing to be randomized to the no intervention arm, and participants’ overall impression of the study. Compliance to treatment will be assessed in group A via capsule count and through a standardized compliance questionnaire at each follow-up visit. Barriers to adherence to progesterone supplementation, and to study follow-up visits, will be assessed using standardized questionnaires.

Exploratory outcomes include (a) changes in serum/urine progesterone levels during pregnancy, (b) correlation of serum/urine progesterone levels with birth weight, birth weight percentile, and gestational age at birth, (c) the relationship between degree of adherence to progesterone supplementation with birth weight, birth weight percentile, and gestational age at birth, (d) the distributions of birth weight and gestational age at birth, including low birth weight (< 2500 g) and small for gestational age births, (e) serum and cell levels of sex steroids, angiogenic, vasoactive, and inflammatory factors, and factors associated with placentation, placenta dysfunction, preterm delivery, and fetal growth restriction, (e) placenta histology, and (f) serum PI drug concentrations.

This study will also provide information on the standard deviation of the change in progesterone levels, and the intra-patient correlation coefficient among repeated progesterone levels during pregnancy to inform the design of a larger RCT.

### Treatment allocation, randomization, and blinding

Participants who consent to the study will be randomized in a 5:3 fashion into two open-label arms: group A (treatment arm) or group B (no-treatment arm). The allocation sequence will be generated in advance by a statistician. Upon obtaining informed consent, study personnel will log into the web-based program to determine the participant’s treatment assignment.

While the participant and the treating physician will not be blinded, the laboratory personnel performing the biochemical and morphological evaluations will be blinded ensuring unbiased evaluation. Study outcomes such as birth weight and gestational age are also objective measures.

### Participant timeline and monitoring

Every participant will be screened and enrolment will occur between GW16 to GW24. The rationale for this study initiation range is threefold: (1) it enables us to capture a larger group of eligible women, (2) it permits progesterone exposure during the time period where most fetal growth occurs (late second and third trimesters), and (3) it omits progesterone exposure during the first trimester where increased risk for hypospadias has been reported [49]. Participants will be followed monthly during their pregnancy, at delivery, and until their exit visit at 4 to 8 weeks post-delivery. During this time, information will be collected from participants’ routine blood work, urine sampling, and prenatal ultrasounds conducted as per standard of care.

During the screening visit, the study is introduced and consent is sought if the woman meets study inclusion and exclusion criteria. A screening log is kept, and the demographics of eligible participants who do not consent to the study are collected. Study personnel collect information on the reasons why a potential participant may have declined participation. Eligible women at less than 16 weeks gestation are screened, but consent and enrollment are deferred to GW16.

For the baseline visit, women undergo a full physical examination. Additional blood and urine samples are collected for assessing progesterone levels and meeting exploratory objectives (i.e., analyzing PI drug concentrations, sex steroids, angiogenic, vasoactive, and inflammatory factors and factors associated with placentation, placenta dysfunction, preterm delivery, and fetal growth restriction). Similar blood and urine sample collection occurs at 1 week after the baseline visit, at GW25–GW28, and at GW33–GW36. During the baseline visit, demographics and relevant medical history are collected by chart review and from the participant as required. Participants are then randomized into either group A or group B. After being appropriately educated on how to self-administer progesterone capsules intravaginally, participants in group A begin progesterone supplementation (administered as two capsules of Prometrium 100 mg) nightly from GW16–GW24 until GW36.

Follow-up visits occur monthly for both study groups. Participants undergo a targeted clinical evaluation. Assessment of safety includes clinical observation and monitoring of laboratory measurements. Symptoms, physical exam findings, and laboratory findings are graded based on the DAIDS Table [48]. At each monthly visit, all participants complete a questionnaire to report any adverse events they may have experienced in the past month. For group A, study medication compliance is assessed at each monthly visit through a capsule count. A compliance questionnaire captures how frequently group A participants have been taking the progesterone and a second adherence questionnaire is provided if participants miss dose(s) to record the reason(s) they missed their medication. If any of the participants miss a follow-up visit, a questionnaire records reasons why they missed their appointment.

At delivery, a clinical and laboratory evaluation of the infant (e.g., birth weight, gestational age at birth, sex, umbilical cord gas results, and Apgar score) is performed according to standard of care. Maternal blood, cord blood, placenta, umbilical cord, and membrane tissue samples are also collected to meet exploratory objectives.

An exit visit occurs 4–8 weeks post-delivery. During this visit, participants undergo a targeted clinical evaluation and complete a short exit questionnaire that allows participants to reflect on their involvement in the study and provide suggestions for the study moving forward with a larger RCT.

### Early termination

Participation in the study will be terminated early if: the participant refuses further treatment and/or follow-up evaluations; the study staff or participant’s medical provider determines that further participation in the study would be detrimental to the participant’s or her fetus’ health or well-being; the participant is non-compliant with the study requirements in a manner that is either detrimental to her health or interferes with the validity of the study results; or the sponsor terminates the study.

### Data recording and adverse event reporting

Data recording will occur using a study case report form (CRF). Data from the CRF will be consolidated into a secure, password-protected internet-enabled database with built-in quality checks.

Any AE that occurs between the time a study participant signs the informed consent form and the time she departs the study at the end of the final follow-up visit (or at the time of early discontinuation from the study for any reason) will be captured and recorded. The start date, the stop date, the severity of each reportable event, and the judgment of the AE’s relationship to the study medication/intervention will also be recorded. All serious adverse events (SAEs) occurring during the course of the study will be reported to the project manager within 24 h of the site becoming aware of the event. Adverse events that had previously been reported by the study subject will also be reassessed for duration, intensity, and possible reoccurrence. All AEs and SAEs will be followed until resolution or until the investigator and the clinical/medical monitor are in agreement that the AE/SAE has stabilized and no more follow-up is required. If an SAE is ongoing at the time a participant discontinues/completes the study, the SAE will be followed until the investigator agrees that the event is satisfactorily resolved, becomes chronic, or that no further follow-up is required.

A Data and Safety Monitoring Board (DSMB) will review the AE/SAE reports submitted by the sponsor/central study coordinator. The DSMB might conclude the study early if unacceptably high rate of AE/SAE is observed.

### Participant rights and confidentiality

Participants will be identified only by means of a coded number specific to each study participant. All participant-related information including CRFs, questionnaires, blood samples, etc. will be kept strictly confidential. All records will be kept in a secure, locked location, and only research staff will have access to the records.

At the screening visit, a consent section separate from the main study consent form asks participants whether their collected samples can be banked and made available for future research. No genetic testing will be performed on these specimens.

### Sample size

This is a pilot study to determine the feasibility of a larger-scale RCT. The sample size of 40 participants was chosen as a feasible number to enroll within a reasonable period of time and to provide sufficient information on the enrollment rate, the acceptability of the intervention, and safety concerns prior to the launch of a larger RCT. Finally, this study will provide estimates of standard deviation and intra-participant correlation coefficients required to calculate the sample size of the larger RCT.

### Statistical analysis

The primary endpoint is the proportion of enrolled participants out of all eligible patients. Qualitative information on the reasons to decline participation will be collected and summarized.

Secondary endpoints include the safety of progesterone supplementation during pregnancy for HIV-positive women as measured by the frequency and severity of AEs in group A and group B. Compliance to progesterone supplementation will be assessed in group A by the proportion of missed doses out of the total prescribed doses per participant. The acceptability of the intervention and barriers to adherence to progesterone supplementation will be recorded for participants in group A. For both group A and B participants, barriers to appointment attendance will be captured and acceptability of the entire study will be assessed in an exit questionnaire.

The relationships between progesterone levels (serum or urine) at baseline, 1 week post-baseline, GW25–GW28 or GW33–GW36 and birth weight, birth weight percentile, and gestational age at birth will be assessed with Spearman’s rank correlation. Serum/urine progesterone levels will be compared between women with and without an AE/SAE using descriptive statistics, if sufficient numbers of events occur. The levels of biomarkers will be compared between the two arms at baseline, 1 week post-baseline, GW25–GW28, GW33–GW36 and delivery visits using descriptive statistics and graphical methods. Qualitative assessment of placenta morphology will be performed blinded to the arm allocation and birth outcome.

Although, this pilot study is not powered for subgroup analyses, qualitative assessment of the impact of race, age, previous adverse pregnancy outcome on enrollment into study, acceptability of progesterone, and adherence will be conducted where possible.

## Discussion

Progesterone supplementation could be of benefit to neonatal health in the context of HIV-positive pregnancy. Progesterone is an essential pregnancy steroid hormone produced by the corpus luteum early in pregnancy and by the placenta starting at 8 weeks of gestation. In the first trimester, progesterone is critical to the maintenance of early pregnancy playing a role in implantation and placenta formation [50, 51]. Later in pregnancy, progesterone is important in maintaining uterine quiescence by limiting production of stimulatory prostaglandins and inhibiting the expression of contraction-associated proteins within the myometrium. Progesterone also has anti-inflammatory effects, which are thought to help maintain the fetal allograft [52]. Functional withdrawal of progesterone activity at the uterus is associated with the onset of labor. Low progesterone levels early in pregnancy have been associated with miscarriage, while low levels in the second and third trimesters have been associated with placental abnormalities, prematurity, fetal distress, fetal growth restriction, and preterm labor [53–56].

Multiple trials on the use of progesterone for the prevention of recurrent preterm birth have been performed in the general population. Progesterone use was associated with longer pregnancy duration and higher mean birth weight [53, 57–62] in most but not all studies [63]. Meta-analyses [44–47, 64] have concluded that progesterone supplementation is protective against recurrent preterm birth, reducing the risk by about 30 %. Neonatal morbidity measures were also favorable with progesterone supplementation although without reaching significance. Meta-analysis of RCTs of progesterone supplementation during pregnancy revealed a significant reduction over placebo in perinatal mortality (six studies, 1453 women), and preterm birth less than 34 weeks (five studies, 602 women). Additionally, significant reductions in preterm birth less than 37 weeks, infant birth weight less than 2.5 kg, use of assisted ventilation, neonatal death, necrotising enterocolitis, and admission to neonatal ICU were also reported [43].

Multiple studies including RCTs and meta-analyses have found no evidence of safety concerns with the use of progesterone for the prevention of preterm birth [41, 43–46, 59–64]. Recognized maternal side effects related to progesterone therapy are headache, nausea, breast tenderness, and coughing [43, 65]. Oral administration of progesterone may lead rarely to impaired biliary excretion and elevated transaminase activity [66, 67]. However, progesterone given vaginally avoids hepatic-first pass effect minimizing the risk of hepatic dysfunction. Natural progesterone has been used in pregnancy without demonstrated effect on fetal development or on the risk of congenital abnormalities [41, 43–46]. There is a possible increased risk of hypospadias in male offspring exposed to exogenous progestins, but the risk is limited to exposure prior to 11 weeks of gestation [49], and we will not be administering progesterone until after gestational week 16. A single retrospective study showed an increased risk of gestational diabetes in women treated with 17alpha-hydroxyprogesterone caproate compared to control subjects [68], but this was not demonstrated in multiple RCTs. The OPPTIMUM study, the largest randomized trial of vaginal progesterone for prevention of preterm birth in women at high risk to date, demonstrated that progesterone had no significant long-term harm on outcomes in children at 2 years of age [69].

In 2008, the Society of Obstetricians and Gynecologists of Canada (SOGC) recommended progesterone supplementation, administered intravaginally (200 mg) for women with a history of previous preterm birth and for women with a short cervical length [41]. In 2011, the Food and Drug Administration (FDA) approved progesterone supplementation, specifically hydroxyprogesterone caproate injections, to reduce the risk of recurrent preterm birth in women with a singleton pregnancy who have a history of at least one prior spontaneous preterm delivery [70]. Despite these recommendations progesterone has not been approved for this use in Canada, and in this setting, its use is considered off label.

In conclusion, progesterone supplementation is used to prevent preterm delivery in high risk, pregnant women; therefore, it is possible that it may also decrease the risk of adverse birth outcomes in pregnant women living with HIV. This pilot study will provide data to determine the feasibility of a larger RCT and inform its study design. It is anticipated that the assessment of progesterone supplementation as a feasible, safe, and acceptable intervention will inform future clinical care and research to improve neonatal health in the context of HIV-positive pregnancies.

## Abbreviations

AE, adverse event/adverse experience; AIDS, acquired immunodeficiency syndrome; ATZ, Atazanavir; cART, combination antiretroviral therapy; CRF, case report form; DAIDS, division of acquired immunodeficiency syndrome, US National Institutes of Health; DRV, darunavir; DSMB, data and safety monitoring board; FDA, Food and Drug Administration of the US Department of Health and Human Services; GCP, good clinical practice; GW, gestational week; HIV, human immunodeficiency virus; ICF, informed consent form; ICH, International Conference on Harmonisation; ITT, intent-to-treat; LPV, lopinavir; N, number (typically refers to participants); NNRTI, non-nucleoside reverse transcriptase inhibitor; PI, protease inhibitor; PI/r, protease inhibitor boosted with ritonavir; RCT, randomized controlled trial; REB, research ethics board; SAE, serious adverse event/serious adverse experience; SGA, small for gestational age; SOP, standard operating procedure; WHO, World Health Organization
