# Erratum to: Progesterone supplementation for HIV-positive pregnant women on protease inhibitor-based antiretroviral regimens (the ProSPAR study): a study protocol for a pilot randomized controlled trial

**DOI:** 10.1186/s40814-017-0135-x

**Published:** 2017-04-28

**Authors:** Kaitlin Siou, Sharon L. Walmsley, Kellie E. Murphy, Janet Raboud, Mona Loutfy, Mark H. Yudin, Michael Silverman, Noor N. Ladhani, Eszter Papp, Lena Serghides

**Affiliations:** 1 Toronto General Research Institute, Toronto, Canada; 2 Toronto General Hospital, Toronto, Canada; 3 University of Toronto, Toronto, Canada; 4 Mount Sinai Hospital, Toronto, Canada; 5 Maple Leaf Medical Clinic, Toronto, Canada; 6 Women’s College Research Institute, Toronto, Canada; 7 St. Michael’s Hospital, Toronto, Canada; 8 St. Joseph’s Health Care London, London, Canada; 9 University of Western Ontario, London, Canada; 10 Sunnybrook Health Sciences Centre, Toronto, Canada

## Erratum

After publication of the original article [1], it came to the authors’ attention that a significant contributor to the protocol, Dr Eszter Papp, had not been included as a co-author.

Dr. Papp contributed to the conception, design, and initial draft of the study protocol, so the Author contributions section of the article should be revised as follows:

**Author contributions**

All authors contributed to the development of the trial protocol and approved the final manuscript. KS led the development of the operations manual and standardization of data collection, undertook initial drafting of the manuscript, and is involved in the overall coordination of the multi-site study. LS, SW, and KM conceived the study, obtained funding, drafted the initial protocol, and are responsible for the communication of the study results. Guidance on clinical data, participant examinations, and study procedures were provided by SW, KM, ML, and MY. JR contributed to the design of this pilot study. SW, KM, ML, MY, NL, and MS led the study at the site responsible for patient recruitment, follow-up, and clinical evaluation. EP contributed to the conception, design, and initial draft of the study protocol.

Dr. Papp has no conflicts of interest to declare.
